# Long COVID in ARDS Survivors: Insights from a Two-Year-Follow-Up Study After the First Wave of the Pandemic

**DOI:** 10.3390/jcm14061852

**Published:** 2025-03-10

**Authors:** Judit Aranda, Isabel Oriol, Núria Vázquez, Karim Ramos, Romina Concepción Suárez, Lucía Feria, Judith Peñafiel, Ana Coloma, Beatriz Borjabad, Raquel Clivillé, Montserrat Vacas, Jordi Carratalà

**Affiliations:** 1 Department of Internal Medicine, Complex Hospitalari Moisès Broggi, 08970 Sant Joan Despí, Spain; 2 Department of Internal Medicine, Consorci Sanitari Alt Penedès-Garraf, 08720 Vilafranca del Penedès, Spain; 3 Infectious Disease Department, Hospital Universitari de Bellvitge, 08907 L’Hospitalet de Llobregat, Spain; 4 Department of Research, Bellvitge Biomedical Research Institute (IDIBELL), 08907 L’Hospitalet de Llobregat, Spain; 5 Center for Biomedical Research in Infectious Diseases Network (CIBERINFEC), Instituto de Salud Carlos III, 28029 Madrid, Spain; 6 Clinical Science Department, Faculty of Medicine, University of Barcelona, 08007 Barcelona, Spain; 7 Statistics Advisory Service, Bellvitge Biomedical Research Institute (IDIBELL), 08907 L’Hospitalet de Llobregat, Spain; 8 Department of Microbiology, CLILAB Diagnòstics, 08720 Barcelona, Spain; 9 Department of Psychiatry and Psychology, Complex Hospitalari Moisès Broggi, 08970 Sant Joan Despí, Spain

**Keywords:** long-term outcomes, sequelae, COVID-19, SARS-CoV-2, ARDS, long COVID

## Abstract

**Objectives:** To compare the health status, exercise capacity, and health-related quality of life (HRQoL) in survivors of COVID-19-associated acute respiratory distress syndrome (ARDS) at 8, 12, and 24 months post-diagnosis. **Methods:** We conducted a prospective, single-center follow-up study embedded within a larger multicenter cohort of adults with COVID-19 who required hospital admission. Eligible participants underwent clinical interviews, physical examinations, chest radiography, and the 6-min walk test (6MWT). Standardized scales were used to assess post-traumatic stress disorder (PTSD), anxiety, depression, and HRQoL. **Results:** Out of 1295 patients with COVID-19, 365 developed ARDS, of whom 166 survived. After excluding deaths and loss to follow-up, 95 patients were monitored for 24 months. Over 60% of patients had persistent symptoms, though significant improvements were recorded in quality of life and physical recovery. More than 70% recovered their previous physical capacity, but 15% did not return to their usual lifestyle habits. Symptoms such as arthralgia and fatigue decreased, but cognitive issues, such as memory loss and insomnia, persisted. Radiological improvements were noted, although pulmonary function remained impaired. The prevalence of PTSD and anxiety decreased, while depression remained stable at around 30%. **Conclusions:** Long COVID continues to impose significant physical, mental, and social challenges. Symptoms like fatigue and anxiety have a profound impact on daily life. Strategies are urgently needed to help patients regain health and resume their normal lives.

## 1. Introduction

The coronavirus disease 2019 (COVID-19) pandemic has unleashed a global crisis of unprecedented scale, ravaging public health, upending societies, and crippling economies, marking it as one of the most profound catastrophes humanity has faced in recent decades. In the early stages of the pandemic, studies focused on determining suitable clinical management and treatment of acute COVID-19 and on its prevention [1,2,3,4,5], while in the second half of the pandemic and during the post-pandemic period, significant progress has been made in understanding the long-term sequelae of the disease. The scientific evidence compiled drew attention to a number of physical, functional, mental and quality of life-related symptoms associated with these long-term sequelae [6,7,8,9]. Long COVID is a complex condition with symptoms lasting four weeks or more after a SARS-CoV-2 infection, involving multiple systems and varying in terms of risk factors, causes, and progression [6,10].

It is currently estimated that long COVID affects at least 65 million people worldwide with conservative projections suggesting that 10% of those infected develop persistent complications. The actual number may be much higher due to the presence of numerous undocumented COVID-19 cases globally; indeed, depending on the cohorts studied, prevalences of up to 50–70% have been reported [11,12,13]. Additionally, patients who suffered acute respiratory distress syndrome (ARDS) during the acute phase of the disease or who required admission to the intensive care unit (ICU) are known to have an increased risk of presenting long COVID [13].

Even though five years have passed since the beginning of the pandemic, long COVID and its management continue to pose a multitude of unanswered questions with significant health, social, and economic repercussions. The limited studies on long-term COVID-19 outcomes beyond 12 months of follow-up have generally included patients with varying disease severity with less emphasis on those most severely affected individuals.

To shed light on these gaps in our knowledge, this prospective study aims to analyze and compare outcomes at 8, 12, and 24 months of follow-up in patients who developed ARDS due to COVID-19 and survived hospitalization. The study focuses on clinical symptoms, exercise capacity, psychiatric disorders, and long-term health-related quality of life (HRQoL).

## 2. Materials and Methods

### 2.1. Study Design

A prospective follow-up study was conducted at a single center within the larger multicenter COVID-MetroSud cohort, which encompassed all adults who required hospitalization for COVID-19 throughout the first wave of the pandemic. This study took place at Complex Hospitalari Moisés Broggi, which is a 350-bed public hospital serving a population of 425,000 in Barcelona, Spain. Follow-up assessments were performed at approximately 8, 12, and 24 months post-discharge.

Our study included adults (>18 years) hospitalized with severe SARS-CoV-2 pulmonary infection between 28 February and 15 April 2020. Only patients who developed ARDS during hospitalization, were discharged alive, and completed all three follow-up visits were eligible. Exclusion criteria included institutionalization, hospitalization during the follow-up period, refusal to involve, failure to establish contact after three attempts, or residence outside the hospital’s catchment area. All participants were thoroughly briefed about the study and granted their written informed consent.

The research followed STROBE guidelines and was approved by the ethics committee of the coordinating center, Bellvitge University Hospital (code HUB-INF-LONG-TERM-METROSUD), in compliance with Spanish regulations. All procedures adhered to the ethical principles of the Declaration of Helsinki (PR140/20).

The data from the initial phase of this study have been previously published [9], and the same methodology has been replicated as in the earlier study to ensure the consistency and comparability of the findings.

### 2.2. Definitions

SARS-CoV-2 infection was confirmed through reverse transcription-polymerase chain reaction (RT-PCR) using a nasopharyngeal swab. The acute phase of infection was defined as the period from the onset of symptoms to hospital discharge. ARDS was diagnosed based on Berlin criteria, which includes the onset or aggravation of respiratory failure following an identified clinical event [14]. It was defined as an oxygen arterial pressure (PaO_2_)/inspired fraction of oxygen (FiO_2_) ratio ≤ 300 mmHg along with radiological findings of diffuse pulmonary opacities not attributable to other causes such as lung edema, masses or atelectasis. The length of hospital stay was defined as the duration between admission and discharge, while the length of ICU stay referred to the total time spent in intensive care with both durations measured in days.

### 2.3. Data Collection, Clinical Evaluation, and Follow-Up

Demographic, epidemiological and sociofunctional information was recorded, as well as comorbidities, clinical data, radiological findings and laboratory test results. Regarding admission to the ICU, we recorded orotracheal intubation, length of stay (in days), complications and treatments received. All data were securely entered into a web-based software platform (REDCap) designed to manage online databases [15].

Patients were assessed at a first follow-up visit between November and December 2020, at a second visit between March and June 2021, and at a final visit between March and June 2022.

**Figure 1:**
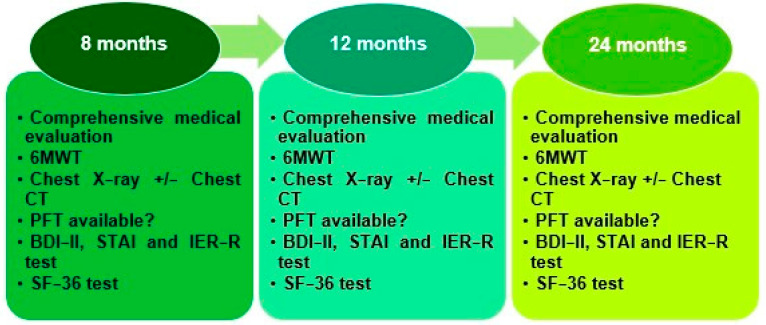
Interventions conducted during 8-, 12- and 24-month follow-up visits.

The following actions were carried out during each of the follow-up visits (Figure 1):

- A thorough medical assessment, including collection of clinical data centered on enduring symptoms, the extent of dyspnea based on the modified British Medical Research Council (mMRC) dyspnea evaluation [16], and a comprehensive physical examination. Patients were additionally inquired about their ability to return to their usual physical and occupational activities as well as whether a close family member had passed away due to COVID-19.
- A six-minute walk test (6MWT) was completed by all patients who were able to walk unaided in accordance with the criteria set of the Spanish Society of Pulmonology and Thoracic Surgery (SEPAR) [17]. The median peripheral oxygen saturation (SpO2) was measured both at baseline and after completing the 6MWT, along with the distance walked, in meters. The formula provided by Enright et al. [18] was employed to calculate the proportion of meters completed relative to the theoretical maximum. Any factors preventing the completion of the test were documented. Dyspnea at the end of the 6MWT was evaluated according the Borg Rating Scale of Perceived Exertion [19].
- A chest X-ray.
- Data from chest computed tomography (CT) scans and pulmonary function tests (PFTs) were gathered whenever accessible.
- Psychological well-being and quality of life were evaluated through various questionnaires: participants filled out the Beck Depression Inventory, Second Edition (BDI-II) [20] to measure depression, Impact of Event Scale Revised (IES-R) [21] to assess post-traumatic stress disorder (PTSD), and State–Trait Anxiety Inventory (STAI) [22] to evaluate anxiety. To examine health-related quality of life (HRQoL), all participants filled out Version 2 of the Short-Form 36 (SF-36), which is a tool consisting of 36 items organized into eight dimensions. These are categorized into two broad categories: the physical component summary (PCS) and the mental health component summary (MCS) [23].

### 2.4. Statistical Methods

Patient characteristics are shown as the total number of cases and percentages for categorical variables and as means and standard deviation (SD) or median and interquartile range (IQR) for continuous variables. To compare characteristics between asymptomatic and symptomatic patients, Fisher’s exact test or the chi-squared test was applied to categorical variables, while the *t*-test or Mann–Whitney U test was used for continuous variables based on the specific requirements. To examine the trends at the 8-, 12- and 24-month follow-up visits, Pearson’s test or Spearman’s test was estimated. All analyses were conducted using the statistical software R version 4.4.0 for Windows (http://www.R-project.org, The R Foundation).

## 3. Results

**Figure 2:**
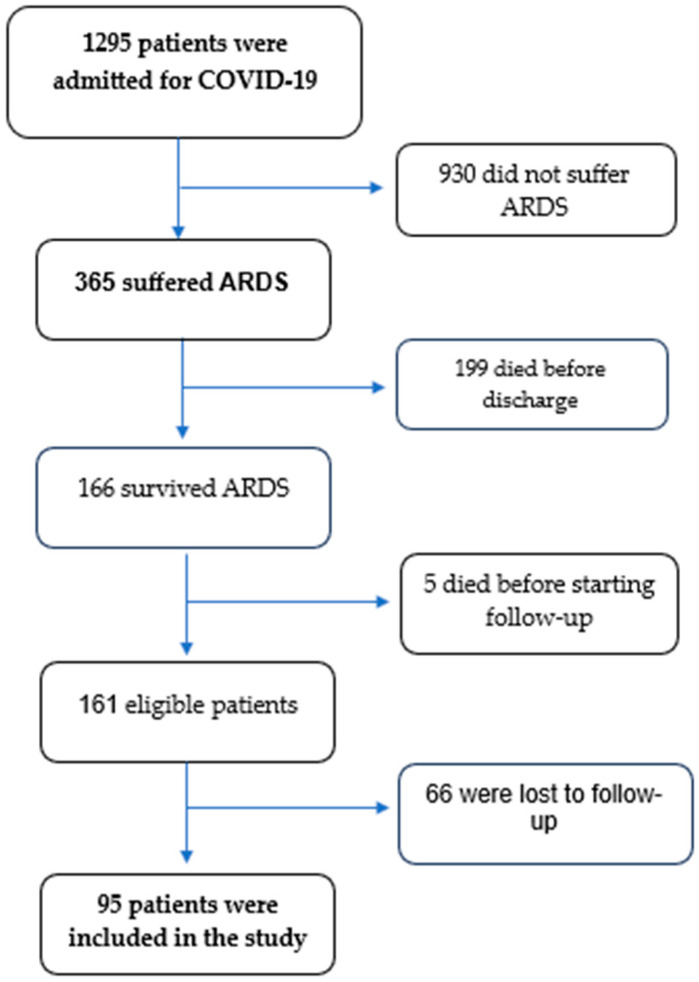
Flowchart illustrating the selection process of patients for the study.

As we already reported in the first report [9], a total of 1295 patients were admitted for COVID-19, of which 365 developed ARDS. Among those in the ARDS group, 199 patients died previous to discharge, resulting in 166 survivors of COVID-19-associated ARDS. Five patients died before starting follow-up and 66 were lost to 24-month follow-up, leaving a cohort of 95 patients. The process of patient selection for the study is presented in Figure 2.

The demographic and clinical characteristics during the acute phase of COVID-19 of patients who remained symptomatic at 24 months and those who did not are shown in Table 1.

After the first positive RT-PCR for SARS-CoV-2, follow-up visits were conducted at a median of 240 days (IQR 232–246) for the 8-month visit, 366 days (IQR 344–398) for the 12-month visit, and 775 days (IQR 762–791) for the 24-month visit.

Table 2 shows the evolution of persistent symptoms throughout follow-up. A significant decrease in symptom persistence was obtained at 24 months (*p* = 0.002), although more than 60% of patients remained symptomatic. Dyspnea was one of the most frequent persistent symptoms and did not present significant changes over the course of follow-up, while arthromyalgia and moderate to severe asthenia were particularly frequent at 8 months of follow-up with a significant decrease at two years (*p* < 0.001 and *p* < 0.001 respectively). The persistence of headache and paresthesia also showed a significant fall after two years. Regarding cognitive sequelae, the prevalence of both subjective memory loss and subjective lack of concentration remained above 40% and the prevalence of insomnia remained above 30% at 8, 12 and 24 months without presenting significant differences. Furthermore, at two years after admission for COVID-19, 84% of the patients had returned to their usual life (*p* < 0.001), and more than 70% had recovered their pre-admission physical exercise capacity (*p* < 0.001).

Table 3 presents the outcomes of the clinical tests. Regarding the 6MWT outcomes, baseline SpO_2_ was similar at all follow-up visits, around 96–97%, whereas the decrease in saturation of ≥4 points after performance of the test fell significantly from 33% at the first visit to 12% at the 24-month follow-up (*p* = 0.004). Likewise, the percentage of patients who covered less than 80% of the theoretical predicted length in meters, adjusted for age, fell significantly from 52% to 32% at two years (*p* = 0.002). Regarding radiological findings, the percentage of normal chest radiographs increased significantly from 45% at 8 months to 76% at 24 months (*p* < 0.001). The most frequent finding in pathological chest radiographs was bilateral interstitial infiltrate. In chest CT, the prevalence of ground glass opacity fell from 37% at 8 months to 10% at 24 months (*p* = 0.102), while fibrosis rose from 11% to 20% (*p* = 0.131). Regarding PFT results, a reduction in carbon monoxide diffusing capacity (DLCO) below 80% of the theoretical level is observed in almost 70% of cases at the 8-month follow-up with no significant changes between the three follow-up visits.

Table 4 summarizes the results of the mental health evaluation, exposing a significant fall in the prevalence of PTSD from 83% at 8 months to 43% at 2 years (*p* < 0.001) and in anxiety status from 54% to 33% (*p* = 0.015). However, the prevalence of depression remained stable at around 30% (*p* = 0.916).

**Figure 3:**
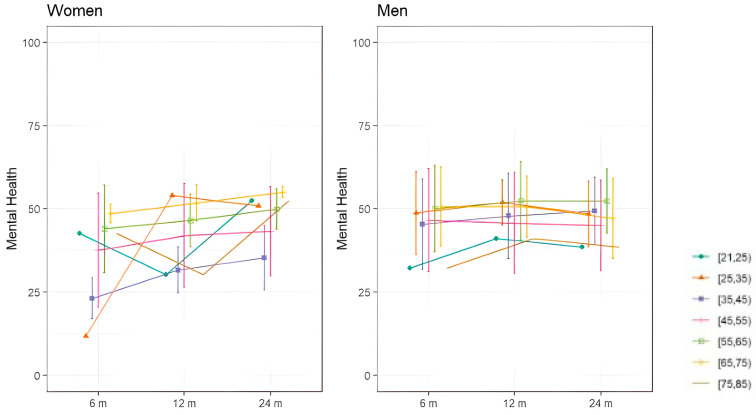
Mean SF36 mental health dimension scores of COVID-19 survivors with ARDS shown by sex in different age groups.

**Figure 4:**
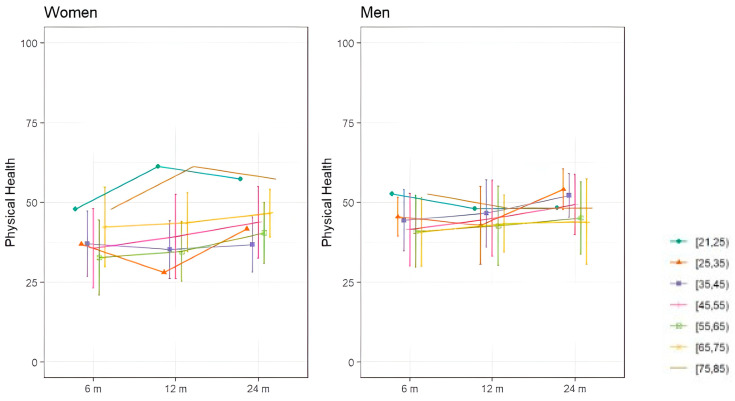
Mean SF36 physical health dimension scores of COVID-19 survivors with ARDS shown by sex in different age groups.

Regarding subjective assessments of quality of life, assessed with the SF36, Figure 3 and Figure 4 show the evolution of mental and physical health perception by sex over the two-year follow-up period. In terms of mental health, women under 35 experienced greater deterioration at 8 months, which progressively improved. Men demonstrated less deterioration in mental health than women. As for physical health perception, women under 25 and those over 75 reported better physical health than other women. Overall, women showed a progressive improvement in both mental and physical health, while men’s health remained relatively stable throughout the follow-up periods.

## 4. Discussion

This prospective study, featuring an in-depth follow-up of patients who developed ARDS during COVID-19 hospitalization, demonstrated a notable reduction in symptom persistence after 24 months. However, over 60% of patients remained symptomatic, the most common symptoms being dyspnea, subjective memory loss, and difficulty concentrating. Additionally, a significant percentage of abnormalities in functional and radiological tests were still present two years after the acute COVID-19 episode, and up to 15% of patients had not yet resumed their usual daily activities.

Although the estimated prevalence of long COVID is much lower than in our cohort [10,24], our data align closely with findings from the German cohort described by Kirchberger et al. [25] and the Spanish cohort reported by Fernández de las Peñas et al. [26]. This difference compared to the estimated prevalence is likely due to a lack of consistent definitions. Nonetheless, like the patients included in our cohort, the evidence suggests that the severity of the initial COVID-19 episode is correlated with the development of persistent symptoms. Likewise, it is now known that infections caused by the Omicron and Delta variants are also more frequently associated with persistent symptoms [10,12,24,27,28].

Similarly, both Kirchberger and Peñas observed prevalence rates of persistent dyspnea comparable to ours (approximately 30–40%) as well as similar rates of subjective memory loss and difficulty concentrating (around 30–50%). In contrast, a Chinese cohort [29] showed a notable decrease in symptom prevalence at the 2-year follow-up, including a reduction in dyspnea from 26% at 6 months to 14% at 24 months. It is important to bear in mind that factors such as access to rehabilitation services and differences in healthcare systems, mental health support, and levels of post-COVID care and monitoring are likely to influence symptom evolution and persistence. Additionally, genetic and environmental differences between populations as well as variations in symptom perception and reporting may affect the extent to which cohorts are comparable.

We observed an improvement in exercise capacity over time with the rate of patients who covered less than 80% of the theoretical predicted length on the 6MWT falling from 52% to 32% at 24 months. Additionally, we noted significant improvements in radiological sequelae. Similarly, chest CT scans showed a reduction in ground-glass opacity and a slight increase in fibrosis. These findings align with results from large cohorts of COVID-19 survivors in China [29,30] as well as the Italian cohort studied by Fumagalli et al. [31]. In agreement with our findings, several studies have described radiological sequelae evolving into fibrosis, particularly in patients with severe COVID-19 and ARDS from different causes [32,33]. In addition, as is to be expected after ARDS, the decrease in DLCO persisted in a high percentage of cases [33].

Interestingly, approximately 30% of patients experienced persistent exercise limitations, and 15% had not resumed their usual lifestyles due to physical or mental sequelae despite significant improvements in mental health at the two-year follow-up. These findings corroborate those of studies reporting reduced anxiety levels in hospitalized and non-hospitalized COVID-19 patients [26,27] and similar exercise limitations in survivors of ARDS [33,34]. The multi-organ impact of COVID-19, including neurological and psychiatric symptoms, highlights the importance of long-term follow-up and rehabilitation programs, as the perceived quality of life progressively improves over time.

A key strength of this study is its prospective approach and the extensive data gathered from a sizable group of patients who experienced severe COVID-19 and survived ARDS. Furthermore, the inclusion of follow-up assessments at 8, 12, and 24 months offers valuable long-term insights into the patients’ progression. However, several limitations should be acknowledged. First, as a single-center study, the findings may not be fully generalizable to other centers or geographic regions. Second, the absence of socioeconomic data for the patients may have influenced the results. Third, in the majority of patients, PFTs and CT scans were not conducted. Fourth, given that all patients in our cohort experienced ARDS, we cannot determine the extent to which this condition itself contributes to long-term sequelae [33,35]. Finally, the vaccination status of the patients under follow-up is unknown, and its potential impact on the prevention of long COVID remains uncertain.

In conclusion, while long-term improvements in clinical symptoms and exercise capacity are observed, the enduring toll on physical and mental health continues to loom large with some patients facing years of persistent challenges. Symptoms such as fatigue, dyspnea, anxiety, and depression cast a long shadow over daily life, while the emotional and social scars of the pandemic’s early days remain deeply etched. Urgent strategies are essential to ensure affected individuals can reclaim their health, restore their quality of life, and return to the normality they deserve.

## Figures and Tables

**Table 1:** Demographic and health features, laboratory results, treatments, and complications occurred throughout the acute episode of COVID-19 of patients who remained symptomatic at 24 months and those who did not.

	Asymptomatic Patients (N = 34)	Symptomatic Patients (N = 61)
**Sex (N; %)**		
Male	26 (76.5)	40 (65.6)
Female	8 (23.5)	21 (34.4)
**Age, years (median; IQR)**	65.5 [55.2–72.8]	61.0 [54.0–71.0]
**Race (N; %)**		
Asiatic	0 (0.0)	0 (0.0)
Caucasian	28 (82.4)	40 (65.6)
Latin	6 (17.6)	16 (26.2)
Black	0 (0.0)	1 (1.64)
Other	0 (4.4)	4 (6.56)
**BMI, kg/m^2^ (median; IQR)**	30.3 [26.7–31.8]	29.2 [25.6–34.0]
**Smoking history (N; %)**		
Smoker	0 (0.0)	2 (3.28)
Former smoker	11 (32.4)	21 (34.4)
Non-smoker	23 (67.6)	38 (62.3)
**Barthel Index score (mean; SD)**	100 (0.0)	99.2 (4.67)
**Comorbidities (N; %)**		
Hypertension	21 (61.8)	23 (37.7)
Diabetes mellitus	10 (29.4)	14 (23.0)
Dyslipidemia	24 (70.6)	29 (47.5)
Atrial fibrillation	2 (5.9)	3 (4.9)
Heart failure	2 (5.9)	0 (0.0)
Chronic kidney disease (CKD) ^1^ of moderate to severe stage	1 (2.9)	0 (0.0)
Chronic respiratory condition	8 (23.5)	17 (27.9)
COPD	1/8 (12.5)	5/17 (31.2)
Asthma	3/8 (37.5)	5/17 (29.4)
OSAS	3/8 (37.5)	7/17 (41.2)
Interstitial lung disease	0/8 (0.0)	1/17 (5.9)
Peripheral vascular disease	5 (14.7)	1 (1.64)
Stroke	2 (5.9)	1 (1.64)
Solid malignancy	2 (5.9)	7 (11.5)
Non metastatic neoplasia	2/2 (100)	7/7 (100)
Metastatic neoplasia	0 (0.0)	0 (0.0)
HIV infection	0 (0.0)	1 (1.6)
Other immunosuppression ^2^	1 (2.9)	0 (0.0)
**Charlson index score** (mean; SD)	3.1 (2.2)	2.7 (1.6)
≤2 points (N; %)	12 (35.3)	32 (52.5)
>2 points (N; %)	22 (64.7)	29 (47.5)
**Treatments (N; %)**		
Lopinavir/ritonavir	1 (2.9)	7 (11.5)
Beta interferon	0 (0.0)	2 (3.3)
Hydroxychloroquine	31 (91.2)	59 (96.7)
Tocilizumab	18 (52.9)	25 (41.0)
Immunoglobulins	0 (0.0)	1 (1.6)
Corticosteroids	26 (76.5)	41 (67.2)
Inhaled corticosteroids	1 (2.9)	11 (18.0)
LMWH	34 (100)	58 (95.1)
**LOS, days (median; IQR)**	29.5 [16.2–46.0]	23.0 [16.0–40.0]
**ICU admission (N; %)**	15 (44.1)	24 (39.3)
Length of ICU stay, days (median; IQR)	20.0 [12.0–30.5]	17.0 [13.0–37.5]
Orotracheal intubation and mechanic ventilation	13/15 (86.7)	21/24 (87.5)
Non-invasive mechanic ventilation	5/15 (33.3)	10/24 (41.7)
**Complications (N; %)**		
Ventilator-associated pneumonia	8/13 (61.5)	5/24 (20.8)
Nosocomial tracheobronchitis	7 (20.6)	6 (9.8)
Heart failure	2 (5.9)	3 (4.9)
Arrhythmia	4 (11.8)	6 (9.8)
Stroke	0 (0.0)	1 (1.6)
Acute coronary syndrome	2 (5.9)	1 (1.6)
PE	7 (20.6)	7 (11.5)
Sepsis	5 (14.7)	5 (8.2)
Mental status abnormalities	4 (11.8)	10 (16.4)
Catheter-related bacteriemia	4 (11.7)	10 (16.4)

Abbreviations: BMI: body mass index; COPD: chronic obstructive pulmonary disease; HIV: human immunodeficiency virus; ICU: intensive care unit; IQR: interquartile range; LMWH: low molecular weight heparin; LOS: length of hospital stay; OSAS: obstructive sleep apnea syndrome; PE: pulmonary embolism; SD: standard deviation. ^1^ Creatinine ≥ 265 μmol/L. ^2^ Solid organ transplantation, hematopoietic stem cell transplantation, chemotherapy, corticosteroid treatment (prednisone> 10 mg/day or equivalent) or neutropenia.

**Table 2:** Clinical characteristics and functional status at 8-, 12- and 24-months follow-up.

	8 Months	12 Months	24 Months
**Days between COVID-19 diagnosis and follow-up visit (median; IQR)**	240 [232–246]	366 [344–398]	775 [762–791]
**Barthel Index score (mean; SD)**	98.4 (6.2)	98.8 (4.8)	98.3 (6.1)
**≥1 persistent symptom (N; %)**	80 (84.2)	81 (85.7)	51 (64.2)
Dyspnea, 0 to 4 points of mMRC			
mMRC = 0	43 (45.3)	51 (53.7)	58 (61.1)
mMRC = 1	32 (33.7)	22 (23.2)	22 (23.2)
mMRC = 2	14 (14.7)	12 (12.6)	11 (11.6)
mMRC = 3	1 (1.1)	5 (5.3)	2 (2.1)
mMRC = 4	5 (4.4)	3 (3.5)	1 (1.4)
Cough	15 (15.8)	15 (15.8)	13 (13.7)
Chest pain	22 (23.2)	20 (21.1)	5 (5.3)
Anosmia	16 (16.8)	17 (17.9)	10 (10.5)
Ageusia	14 (14.7)	12 (12.6)	7 (7.4)
Odynophagia	13 (13.7)	12 (12.6)	6 (6.3)
Asthenia, 0 to 10 points			
<5 points	56 (58.9)	48 (50.5)	75 (78.9)
≥5 points	39 (41.1)	47 (49.5)	20 (21.1)
Arthromyalgia	49 (51.1)	44 (46.3)	14 (14.7)
Headache	32 (33.7)	28 (28.5)	12 (16.9)
Subjective memory loss	41 (43.2)	44 (46.3)	41 (43.2)
Subjective lack of concentration	41 (43.2)	45 (47.7)	44 (46.3)
Insomnia	27 (36.0)	26 (34.7)	24 (32.0)
Paresthesia	23 (24.2)	26 (27.4)	9 (9.5)
**Functional capacity and family impact** **(N; %)**			
Returned to normal activities	54 (56.8)	68 (71.6)	80 (84.2)
Back to work	30/56 (53.6)	33/46 (71.7)	44/52 (80.5)
Resumed physical exercise	42 (44.2)	49 (51.6)	70 (73.7)
Family members affected by COVID-19	46 (48.4)	60 (63.2)	80 (84.2)
Family members deceased due to COVID-19	9 (9.5)	14 (14.7)	14 (14.7)

Abbreviations: IQR: interquartile range; SD: standard deviation; mMRC: modified British Medical Research Council score.

**Table 3:** The 6MWT, radiological tests, and PFT results at 8-, 12- and 24-month follow-up.

	8 Months	12 Months	24 Months
**Total 6MWT: N = 85 (89.5% of patients)**			
Initial SpO_2_, % (median; IQR)	97 [96.0–98.0]	96 [96.0–98.0]	97 [96.0–98.0]
Final SpO_2_,% (median; IQR)	95 (93.0–96.0)	95 (93.0–96.0)	95 (94.0–96.0)
Decrease in SpO_2_, ≥4% (N; %)	28 (32.9)	22 (25.8)	10 (11.8)
Initial or final SpO_2_, <88% (N; %)	4 (4.7)	4 (4.7)	1 (1.2)
Meters completed (mean; SD)	385 (103)	411 (86.8)	438 (82.1)
Completed <80% of theoretical distance in meters (N; %)	44 (51.7)	34 (40.0)	27 (31.8)
6MWT interruption (N; %)	4 (4.7)	3 (3.5)	0 (0.0)
**BORG scale at the conclusion of 6MWT, points (N; %)**			
0–2	66 (78.6)	69 (82.1)	79 (94.0)
3–7	18 (21.4)	0 (0.0)	0 (0.0)
**Radiological findings**			
**Total chest X-ray: N = 84 (88.4% of patients)**			
Days between COVID-19 diagnosis and chest X-ray (median; IQR)	132 [93–246]	292 [182–345]	690 [567–724]
**Normal chest X-ray**	**38 (45.2)**	**51 (60.7)**	**64 (76.2)**
Bilateral interstitial infiltrate	9/46 (19.5)	13/33 (39.7)	15/20 (75.0)
Bilateral alveolar-interstitial infiltrate	4/46 (8.7)	1/33 (3.0)	0/20 (0.0)
Unilateral alveolar infiltrate	1/46 (2.2)	0/33 (0.0)	0/20 (0.0)
Unilateral interstitial infiltrate	1/46 (2.2)	1/33 (3.0)	0/20 (0.0)
**Chest CT (N; %)**	**27 (28.4)**	**42 (44.2)**	**40 (42.1)**
Ground-glass opacification	10/27 (37.0)	7/42 (16.7)	4/40 (10.0)
Consolidation areas	1/27 (3.7)	0/42 (0.0)	0/40 (0.0)
Ground-glass and consolidation	5/27 (18.5)	4/42 (9.5)	1/40 (2.5)
Fibrosis	3/27 (11.1)	4/42 (9.5)	8 (20.0)
PE	0/27 (0.0)	0/42 (0.0)	0/40 (0.0)
**PFTs performed at the three follow-ups**			
PFT available (N; %)	42 (44.2)	29 (30.5)	35 (36.8)
Days between COVID-19 diagnosis and PFT (median; IQR)	190 [140–210]	218 [186–325]	673 [606–726]
FEV1 < 80% (N,%)	10 (23.8)	8 (27.6)	8 (22.9)
FVC < 80% (N,%)	11 (26.2)	12 (41.4)	8 (22.9)
FEV1/FVC < 80% (N,%)	6 (14.3)	2 (6.9)	8 (22.9)
DLCO < 80% (N, %)	28 (66.7)	23 (79.3)	28 (80.0)

Abbreviations: 6MWT: 6-min walk test; Chest CT: chest computed tomography; IQR: interquartile range; mMRC: modified British Medical Research Council score; PE: pulmonary embolism; PFT: pulmonary function test; SD: standard deviation; SpO_2_: peripheral oxygen saturation.

**Table 4:** Results of testing for mental health disorders at 8-, 12- and 24-month follow-up.

	8 Months	12 Months	24 Months
**Total BDI-2 inventory of depression test: N = 92 (96.8% of patients)**			
Minimal or no depression	62 (67.4)	59 (64.1)	65 (70.7)
Mild	7 (7.61)	9 (9.8)	5 (5.4)
Moderate	15 (16.3)	14 (15.2)	15 (16.3)
Severe	8 (8.7)	10 (10.9)	7 (7.6)
**Total IES-R of PTSD test: N = 93 (97.9% of patients)**			
Minimal or no PTSD	16 (17.2)	31 (33.3)	53 (57.0)
Mild	31 (33.3)	28 (30.1)	26 (28.0)
Moderate	6 (6.5)	5 (5.4)	2 (2.2)
Severe	40 (43.0)	29 (31.2)	12 (12.9)
**Total STAI state anxiety test (percentile): N = 89 (93.7% of patients)**			
<75	41 (46.1)	48 (53.9)	60 (67.4)
≥75	48 (53.9)	41 (46.1)	29 (32.6)
**Total STAI trait anxiety test (percentile): N = 88 (92.6% of patients)**			
<75	55 (62.5)	55 (62.5)	66 (75.0)
≥75	33 (37.5)	33 (37.5)	29 (32.6)

Abbreviations and definitions: BDI-2: Beck Depression Inventory; IES-R: Impact of Event Scale Revised, a post-traumatic stress assessment scale; PTSD: post-traumatic stress disorder; STAI: State–Trait Anxiety Inventory.

## Data Availability

Data are contained within the article.
